# Exploring farmer and advisor lameness management behaviors using the COM-B model of behavior change

**DOI:** 10.3389/fvets.2024.1258906

**Published:** 2024-01-17

**Authors:** Beth Clark, Amy Proctor, Niamh Mahon, Lewis Holloway

**Affiliations:** ^1^Centre for Rural Economy, School of Natural and Environmental Sciences, Newcastle University, Newcastle upon Tyne, United Kingdom; ^2^Social, Economic and Geographical Sciences Group, James Hutton Institute, Aberdeen, United Kingdom; ^3^School of Environmental Sciences, Faculty of Science and Engineering, University of Hull, Hull, United Kingdom

**Keywords:** lameness, animal welfare, animal health, cattle, sheep, behavior change

## Abstract

**Introduction:**

This paper applies the COM-B framework to farmer and farm advisor understandings and responses to lameness in sheep, beef, and dairy systems. It reflects on how farmers' and advisors' capability, opportunity, and motivation (COM-B) influence lameness management practices in these farming systems, and considers the interaction between these three factors, and stakeholders' behavior.

**Methods:**

Interviews with 29 farmers and 21 farm advisors in the north of England were conducted. Thematic analysis was undertaken with results categorized in relation to the COM-B framework focusing on barriers and enablers of lameness management. Use of the COM-B model provides a useful means of understanding the underlying behavioral mechanisms that contribute toward the persistence of lameness. This includes the complexities and interactions which hamper implementation of lameness management best practice.

**Results and discussion:**

The findings highlight three key areas to address with interventions to improve lameness management on farm: (1) removing physical and social barriers for lameness management; (2) improving psychological capability and motivation for lameness management; and (3) facilitating relationships and developing communication between farmers and advisors. In particular, the value of exploring both farmer and advisor perspectives on behavior in the animal health context is demonstrated. Future interventions should look to target these three areas to overcome barriers and focus on factors that enable positive lameness practices to occur.

## 1 Introduction

### 1.1 Overview

This paper applies the COM-B framework (1) to research exploring farmer and farm advisor understandings of and responses to lameness in sheep, beef and dairy systems. It reflects on how farmers' and advisors' capability, opportunity, and motivation (referred to as COM-B) influence lameness management behaviors in these systems. It also considers these factors in the interactions between farmers and their advisors and how this might influence behavior toward lameness management. The paper begins with a review of literature on lameness, lameness management and behavior change before introducing the methodological approach. It then presents the findings in relation to how capability, opportunity and motivation relate to barriers and enablers of lameness management for farmers and their advisors, and how these can be targeted for effective lameness management. The application of behavior change frameworks to lameness management is highlighted across different stakeholder groups. The paper concludes by highlighting three key areas for changing behavior. Firstly, removing physical and social barriers to opportunities for lameness management. Secondly improving farmers' and advisors' psychological capability and motivation for lameness management. Thirdly facilitating relationships and developing communication between farmers and advisors.

### 1.2 Background

Lameness refers to conditions of the legs and feet of an animal that affect its gait (2). Lameness can be caused by disease, management, environmental or physical factors or a combination of these (3, 4). Lameness in cattle and sheep is a significant health and welfare issue and within the UK has been cited as one of the top endemic conditions of concern in the beef, dairy and sheep sectors (5–8). Lameness leads to reduced animal productivity and additional economic costs through increased farmer/worker time and treatment expense (3, 9–12).

In the UK, both national and farm-scale estimates of lameness levels in cattle and sheep vary (13–16). In dairy cattle it is thought that around 30% of the UK herd are lame at any one time (11), with prevalence on farm estimated between 6.6 and 35% [(17) in (16)]. Lameness is thought to affect 90% of sheep flocks (13). Although lameness levels have reduced to 4.9% in 2013 (3), they are still higher than the <2% target introduced in 2011 by the Farm Animal Welfare Council (18). Whilst there is a smaller body of research in the UK for beef cattle (19), one study found that UK mean farm level prevalence of lameness was 8.3% for finishing cattle and 14.2% for suckler cows (14).

Given its multifactorial nature, lameness can be complex to prevent and treat. Research has provided insight into the causes and subsequent management strategies surrounding lameness (4, 20, 21). In the UK, this has led to the development of several recommended tools for lameness management, such as the 5-point plan for sheep (22) as well as the Agriculture and Horticulture Development Board's Healthy Feet and Healthy Feet Lite Programmes for cattle (4, 21). These have been shown to be successful in providing a framework for reducing lameness prevalence on farm. However, despite their success, best practice advice is not always followed (23–25). Lameness therefore presents a persistent problem, with better ways of understanding and managing this endemic health issue urgently required (26). The management of lameness, and many risk factors associated with the condition, are under the control of farmers (14). A reduction in lameness often requires farmers to change their behavior by adapting their existing practices or resources (24). Often this is advised by veterinarians and other advisors such as foot trimmers and specialist lameness consultants (27). Understanding the role farmers and their advisors play in treatment and prevention is important (2). This includes barriers to the adoption of recommended lameness management practices, as well as the factors that facilitate or motivate change (28).

### 1.3 Challenges in lameness management

Several reasons why farmers do not follow recommended guidelines on lameness prevention have been identified (24, 29). Advice may not always be practical to implement, due to competition with other farm tasks, or issues associated with time constraints, staffing or space availability. Financial considerations also play a role, with larger farms thought to be more likely to invest in protocols for biosecurity, including veterinary services and equipment costs (30). Some practices are considered too expensive by some farmers, such as antibiotic use (31). Farmers may also have a lack of confidence in their ability to implement new practices (32), or be reluctant to change their current management techniques (31, 33). In addition, factors influencing farmer behavior toward the adoption of recommended practice have also been identified. These include extrinsic factors [such as group norms (30)], and intrinsic factors [such as motivation, emotion and personality (13, 32)], sociodemographic and household characteristics, farm business structure (34, 35) and those related to the “good farmer” identity (36). Furthermore, there is evidence to suggest that perceptions and understandings of lameness present particular challenges for its effective management. A lack of awareness of lameness (37), inaccurate perceptions of lameness levels (38), or underestimated levels all make early identification and treatment difficult (24, 39–41). Moreover, for some farmers, lameness is simply accepted as an inevitable part of farming process (29, 32, 42).

Whilst lack of knowledge of animal care and behavior by animal caregivers has been identified as an important welfare issue (43), it may not always be a lack of knowledge about recommended lameness practice that may be contributing to its persistence (24, 42, 44, 45). Rather, there is a need to better understand the wider factors which may influence how this knowledge is applied in specific farm contexts, and how decision making surrounding animal health and welfare is made on farm (44).

Lam et al. (27) argue that to influence behavior in relation to animal health, the farmer or stockperson must have the right knowledge about optimal practices in relation to lameness and they need to be motivated enough to adopt those practices (46). Understanding the factors motivating behavioral change is important for encouraging efforts to drive lameness control (2), and has received increasing attention in relation to encouraging farmers to make changes of various kinds (47). Farmers' motivations for lameness prevention and treatment may vary, and can include animal welfare concerns, economic considerations such as animal productivity, and social wellbeing and morale concerns for themselves and their staff (4, 48).

### 1.4 The role of veterinarians in behavior change

Whilst veterinary medicine has tended to focus on knowledge generation and transfer in relation to influencing farmer behavior change (49), increasing attention is being paid to motivation and the role of the veterinarian in this. Decision makers on farms vary in their values and motivations, and in the factors that influence their decisions (50, 51). Understanding these and their impact on behavior is important for stakeholders interested in advising farmers and influencing their decision-making (50). There has been a wealth of literature in recent years exploring this. This has focused on motivational interviewing in the dairy sector (52, 53), the changing role of veterinary practice including toward more preventative practice (54), the importance of farmer and advisor relationships (55), and the need to identify “rings of confidence”^1^ (56) or trusted/key sources of information (35). This body of research emphasizes that to enable advisers to best target communications and encourage change in behavior regarding recommended lameness management practices, there is a need to better understand the factors underpinning farmer behavior (55). This includes motivations, individual and farm characteristics, and relationships with others (including farmers and/or advisors).

Behavior change frameworks offer a means to understand these factors and their influence on behavior. These provide a route to identifying more nuanced and effective interventions (50, 57) and are receiving increasing interest in academia, industry, and policy. The utilization of a behavior change framework may provide insights and understandings of why behavior has not changed despite the existence of best practice and tools for managing lameness.

### 1.5 Behavior change frameworks in the management of animal health

Behavioral change research originated in the field of health psychology before its application in an array of different contexts including animal health. Many studies have sought to explore farmer behavior over recent decades, although not all have used a behavioral theory in their study design (55). These studies have explored farmer behavior from a range of different disciplinary perspectives, including psychology and sociological theory (55), and s economics (58). These studies have provided different insights to understandings of behavior and behavior change.

The Theory of Reasoned Action (59) and the Theory of Planned Behavior (60) have been used extensively in relation to human health (61), and increasingly, have been used to explore a broad range of farm management practices (35, 55, 62). This includes a body of research that has led to successful behavior change interventions in stockpersons both on and off-farm, for the improvement of animal welfare (63–65). This research has primarily focused on the attitude-behavioral intention relationship included within the Theory of Reason Action and the Theory of Planned Behavior models (66).

More recently work on behavior change has moved away from utilizing a single framework toward those that are a synthesis of multiple models or frameworks, such as the Transtheoretical Domain Framework (67), Behavior Change Wheel (1) from human health psychology, or the RESET Mindset model from animal health psychology (27). Research by Lam et al. (27) on antibiotic usage with dairy farmers and veterinarians in the Netherlands indicated that for positive behavior change to occur not only did the knowledge of dairy farmers and veterinarians have to improve, but also their mindset^2^ in relation to the subject of change. Using the RESET Mindset model, Lam et al. (27) were successfully able to decrease antibiotic usage behavior through a combination of compulsory and voluntary activities.

This shift toward synthesis models of behavior enables the consideration of a more comprehensive range of factors that might influence human behavior. A detailed understanding of the behavior in question is essential for developing the most effective interventions. A recent review by Biesheuvel et al. (55) into farmers' behavior surrounding cattle diseases identified a large number of constructs or factors that may influence farmer behavior, further emphasizing the complexity of developing responses. Biesheuvel et al. (55) argue that utilizing a model that looks to understand the behavior and its underlying mechanisms is therefore important for identifying the most suitable interventions.

**Figure 1:**
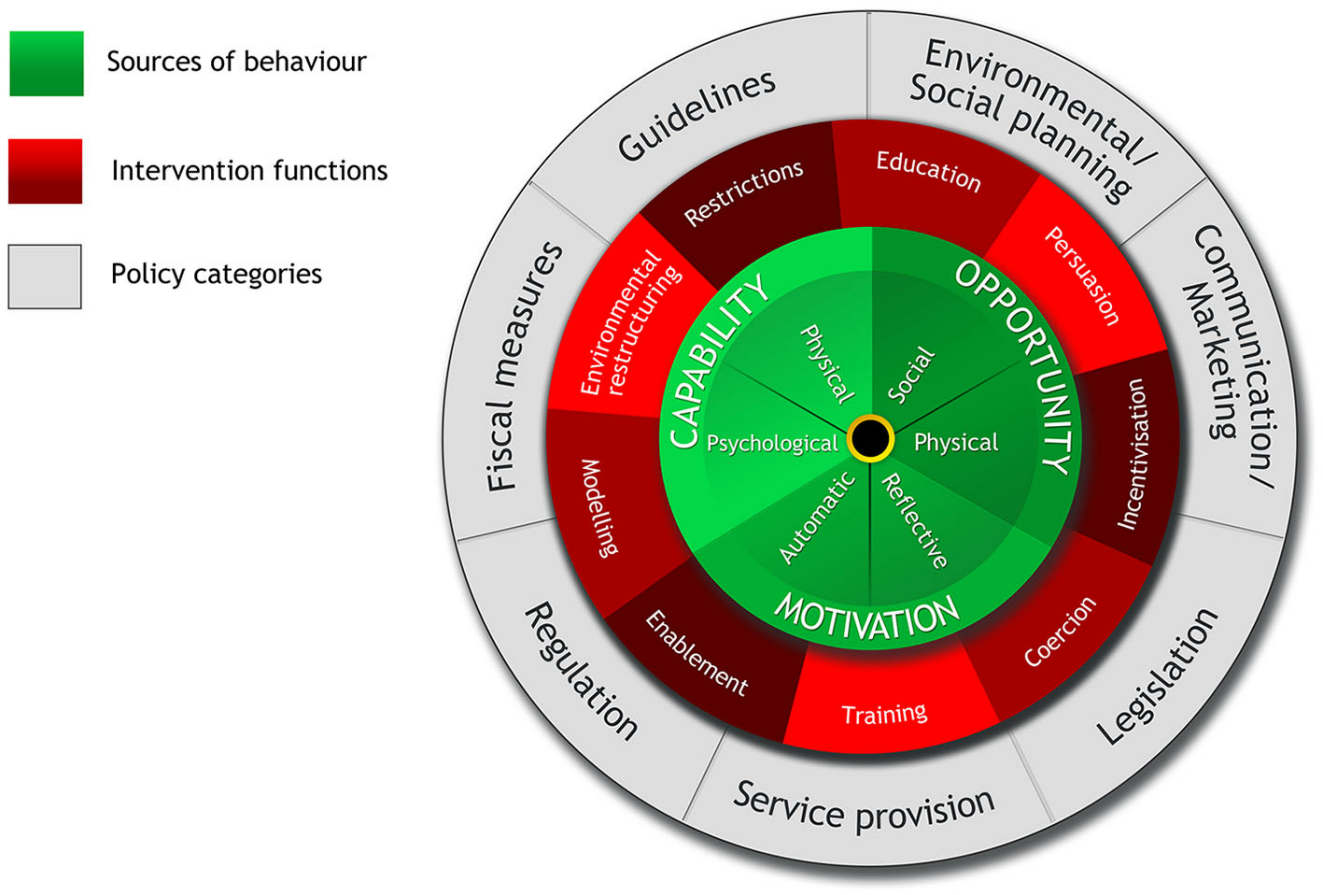
The behavior change wheel (1).

One example of a framework that seeks to understand a given behavior and its underlying mechanisms is the Behavior Change Wheel (BCW) (1). Originating in human healthcare, the BCW is a synthesis of 19 frameworks of behavior change (1) and aims to not only identify influences on behavior change but also the mechanisms of change and subsequently the most suitable interventions. The BCW (Figure 1) is split into three layers. At its core are the “sources” of behavior (capability, opportunity, and motivation). Nine intervention functions are presented in the middle, with the outer layer identifying seven types of policy response that can be used to deliver these interventions. Factored within the BCW is the assumption that behavior is driven by beliefs, perceptions, unconscious biases, mental shortcuts, and physical and contextual environments (68). It thus takes into account both individual and broader contextual and social constructs, offering a potentially more comprehensive framework (55).

Carroll and Groarke (69) argue that the advantage of using the BCW is the simplicity and clarity of the COM-B system at its core. The BCW presents a systematic way of determining which of the three sources of behavior, i.e., capability, opportunity, and motivation, are most likely to need intervention to achieve the change in behavior required. An overview of these central sources are in Table 1. By understanding the behavior in question through the COM-B model, the most suitable intervention options, including the content and implementation of these, can be identified.

**Table 1:** An overview of capability, opportunity, and motivation.

	**Description**	**Types**
Capability	There must be the *capability* to do it: the person or people concerned must have the physical strength, knowledge, skills, stamina etc. to perform the behavior	*Physical*: skills, strength, stamina *Psychological*: knowledge, understanding, psychological skills, strength, or stamina to engage in necessary mental processes
Opportunity	There must be the *opportunity* for the behavior to occur in terms of a conducive physical and social environment: e.g., it must be physically accessible, affordable, socially acceptable and there must be sufficient time	*Physical:* what the environment allows or facilitates in terms of time, triggers, money, other resources, locations, physical barriers etc. *Social:* interpersonal influences, support from others, social cues and cultural norms that influences the way we think about things
Motivation	There must be sufficiently strong *motivation*: i.e., the individual must be more highly motivated to do the behavior at the relevant time than not to do the behavior, or to engage in a competing behavior	*Reflective:* involves plans (self-conscious intention) and evaluations (beliefs about what is good or bad) *Automatic:* processes involving wants, needs, desires, impulses, inhibitions, and reflex responses including habits and emotions

Adapted from Michie et al. (68).

The BCW and COM-B elements, shown in Figure 1, have been applied to studying farmer behavior in a range of contexts including invasive species control (70), farm safety (71), grass measurement (72), antimicrobial stewardship amongst vets (57), the reduction of tail biting on pig farms and BVD management in cattle farmers (73, 74). Carroll and Groarke (69) also provide a wide-ranging review of its potential use in animal welfare and agricultural research. Given the multitude of factors influencing lameness management as highlighted in section 1.3, this study applies the COM-B model to further explore which of these are most influential on behavior. This in turn will enable the most effective interventions to be identified.

Whereas previous research has sought to explore behavioral change frameworks for changing behaviors in either farmers or their advisors (73), consideration of both together is less common (27). Exploring both farmer and advisor perspectives is important given their joint role in managing animal health and welfare, their interactions, and different responsibilities and challenges within this context. Given the persistent challenge of lameness, the aims of this paper are to gain a better understanding of the decision-making process and mechanisms underlying lameness management behavior for farmers and their advisors. This is particularly pertinent given the changes in farming subsidies occurring in England post-Brexit within the Animal Health and Welfare Pathway (AHWP) (75), with lameness featuring as a priority area for sheep and cattle (beef and dairy). Understanding both farmer and advisor behavior is therefore important for ensuring that they are best supported in delivering any new targets.

## 2 Methods

The research presented in this study is part of a larger research project Farm-level Interdisciplinary approaches to Endemic Livestock Disease (FIELD), that explored why endemic livestock disease persist in the UK. In-depth, semi-structured interviews were undertaken with farmers and their advisors. This included a range of advisory professions as outlined in Table 2. Interviewing farmers and their advisors ensured that perspectives from a range of stakeholders involved in lameness management were obtained.

**Table 2:** Participant overview.

**Interviewee**	**Gender**	**Livestock kept**	**Interviewee**	**Gender**	**Role**
Farmer 1	Female	Sheep	Advisor 1	Female	Pharmaceutical representative
Farmer 2	Male	Dairy cattle	Advisor 2	Male	Cattle hoof trimmer
Farmers 3 & 4	Female & male	Beef cattle, sheep	Advisor 3	Male	Cattle hoof trimmer
Farmer 5	Male	Beef	Advisor 4	Female	Veterinary consultant
Farmer 6	Female	Beef cattle, sheep	Advisor 5	Female	Levy board staff
Farmer 7	Male	Beef cattle, sheep, dairy cattle	Advisor 6	Female	Livestock nutritionist
Farmer 8	Male	Beef cattle, sheep	Advisor 7	Female	Vet
Farmers 9 & 10	Female & male	Beef cattle, sheep	Advisor 8	Male	Vet
Farmer 11	Female	Sheep	Advisor 9	Male	Veterinary consultant
Farmer 12	Male	Beef cattle, sheep	Advisor 10	Male	Vet
Farmer 13	Male	Beef cattle	Advisor 11	Female	Vet
Farmer 14	Male	Beef cattle, sheep	Advisor 12	Female	Vet
Farmer 15	Male	Dairy cattle	Advisor 13	Male	Farm consultant
Farmer 16	Male	Dairy sheep	Advisor 14	Male	Veterinary consultant
Farmer 17	Male	Dairy cattle	Advisor 15	Male	Livestock auctioneer
Farmer 18	Male	Dairy cattle	Advisor 16	Female	Vet
Farmer 19	Male	Dairy cattle	Advisor 17	Female	Farm consultant
Farmer 20	Male	Beef cattle, sheep	Advisor 18	Female	Farm consultant
Farmer 21	Male	Beef cattle, sheep	Advisor 19	Female	Assurance scheme assessor
Farmer 22	Male	Beef cattle, sheep	Advisor 20	Male	Vet
Farmer 23	Male	Beef cattle, sheep			
Farmer 24	Female	Beef cattle, sheep			
Farmer 25	Male	Beef cattle			
Farmer 26	Female	Sheep			
Farmer 27	Male	Beef cattle, sheep			
Farmer 28	Male	Sheep			
Farmer 29	Male	Beef cattle, sheep			

Interviews were primarily conducted by BC and NM. Semi-structured interview guides were developed for farmers and advisors respectively (see Supplementary material), informed by the findings of a preliminary survey with farmers (44). This aided in ensuring consistency across interviews whilst also allowing for flexibility of conversation.

All farmers interviewed were based in the north of England and raised one or a combination of sheep, beef, and dairy cattle. Efforts were made to recruit farmers across each group, although due to the lower levels of dairy production in the region, fewer dairy farmers participated. Several advisors were based in and operated out of the north of England, with others practicing at the national, and sometimes international, level. Farmers and advisors were recruited through a range of mechanisms including advertising in farming press, direct contact, and snowballing.

In total 29 farmer interviews and 21 advisor interviews were conducted between September 2019 and March 2021. Research was affected by the COVID-19 restrictions imposed in the UK from 23rd March 2020 (76). As a result, only 11 farmer interviews, and two advisor interviews were conducted face-to-face, with the remainder conducted either virtually (online) or over the phone. All participants provided written and/or verbal consent to take part. Interviews lasted between 60 and 150 min. Ethical approval for the research was granted from Newcastle University's Faculty of Science, Agriculture and Engineering Ethics Committee prior to research commencing (Refs: 15572/2018; 1336/2020).

All interviews were recorded and transcribed by a professional transcription agency. Transcripts were then checked for accuracy by the original interviewer. All transcripts were entered into NVivo (77) where thematic analysis was undertaken (78). To aid analysis a codebook was co-developed by all members of the research team. This was devised during the interview process and through all authors reading and familiarizing themselves with the interview transcripts. Several discussion sessions were then held by the authors to devise, define and review suggested codes. The developed codebook was trialed on both the farmer and advisor interview transcripts by all members of the research team, with each transcript coded by two of the authors independently. Codes were interrogated and content noted against the COM-B framework (see Table 1) to identify data relevant to capability, opportunity and motivation. The material under each COM-B element was then grouped into themes,. These themes were then reviewed to ensure that the included data supported each theme, and that links between the different themes were noted.

## 3 Results

### 3.1 Overview

Lameness was recognized by participants as a threat to animal health and welfare. Participants identified several consequences of lameness and had both internal and external motivations for enacting lameness management behaviors. Their beliefs surrounding lameness management led to farmers and their advisors adopting a range of different practices on farm to identify and treat lame animals and prevent lameness in the first instance.

Whilst the importance of context specific advice and guidance (taking into account both the farm and farmer), was emphasized throughout the interviews, a number of more general themes were also identified. These surrounded lameness management behaviors relating to capability, opportunity, and motivation, Including: the application of knowledge, resource constraints, perceived control over being able to prevent lameness, the importance of relationships, supportive assurance and legislation and the “good farmer” identity (36).

**Figure 2:**
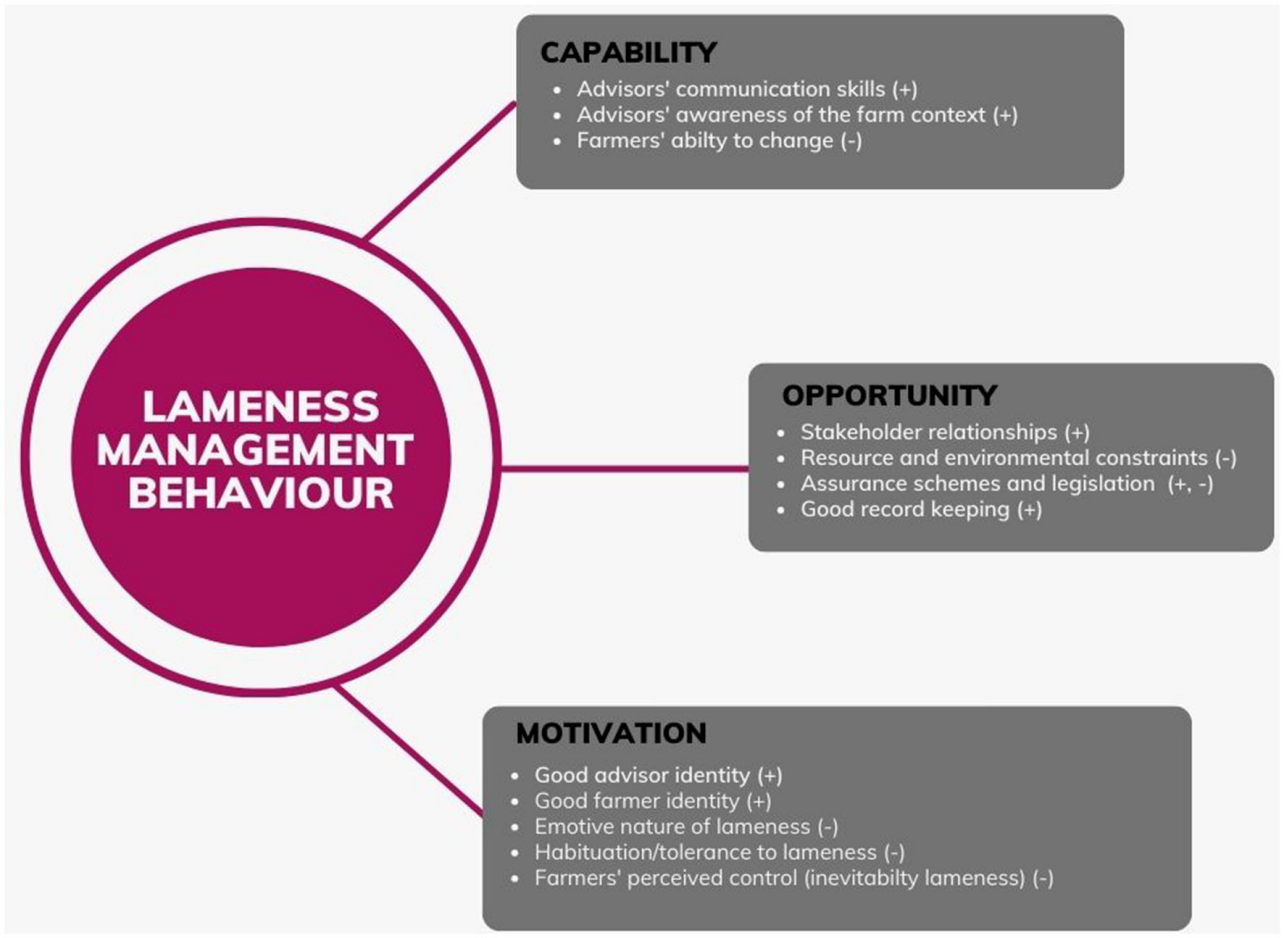
Themes arising from the interviews in relation to capability, opportunity and motivation. + Denotes enablers of lameness management, – denotes barriers to lameness management.

Both farmer and advisor interviews explored several elements of capability, opportunity, and motivation. An overview of the key factors arising from the interviews can be found in Figure 2, which outlines where the main themes arising from the interviews can be positioned in relation to capability, opportunity, and motivation. These themes are presented below, starting with barriers to, then enablers of, lameness management, alongside supporting quotations from the interviews.

### 3.2 Barriers to lameness management

Several barriers to managing lameness were apparent for both farmers and advisors. For farmers, barriers related to psychological capability, their application of knowledge, self-efficacy, ability to change, and lack of motivation because of a habituation/tolerance to lameness and an acceptance of the inevitability of animals becoming lame. The emotive nature of lameness was also raised, with a lame animal potentially a reflection on farmers' management. Lack of physical and social opportunity presented barriers or constraints linked to time, economics, the farm environment, and social pressure via assurance schemes. For advisors, physical and social opportunity were also identified as barriers, with these tied to farmer resource constraints, and barriers to developing relationships.

#### 3.2.1 Capability

Both farmers and advisors had the individual physical capability to manage lameness, although certain tasks were deferred to others, particularly hoof trimmers for cattle. Farmers were asked to describe lameness and its symptoms during the interviews. Farmers could provide definitions and explanations of lameness and articulate what a lame animal is, how they would treat individual lame animals and steps they would take to prevent lameness at a flock/herd level.

“*I footbath them on a regular basis, I do the sheep in two batches, or three batches and they'll all get done alternate Saturday's, near enough. Bring them in, run them through, if there's any to manually treat, treat them and do it that way, just keep on top of it really*.” (Farmer 14)

Several farmers discussed causes, diagnosis and prevention in great detail as illustrated by the quotation below.

“*It's the inability of cows to place equal weight on all their feet, really, and having to compensate from foot problems, whether that's from an external source or a genetic source because it's not always a problem with them. It can be an internal infection within the foot, or it can be an external wound, something actually injured the foot. It can even be a foot trimming that's been over harsh or that's gone wrong, it can be caused by cows being kept in the wrong environment, poor cow roadways and tracks*.” (Farmer 15)

Several advisors highlighted a key problem was persuading farmers to apply this knowledge and understanding of good practice. As one said, “*the application of what we know about lameness is a bigger challenge than the actual problem itself* ” (Advisor 12). It was argued that this gap between possessing knowledge and its practical application led to higher lameness incidence rates. Several factors tied to motivation affected this (see Section 3.2.3) which in turn influenced farmers' ability to change behavior.

#### 3.2.2 Opportunity

Several barriers or constraints denied farmers the physical opportunity to manage lameness effectively, including resource constraints and the farm environment. These could be perceived or actual barriers. As one advisor described, “*They [barriers] have remained unchanged. It's time and money”* (Advisor 9). The time taken to keep on top of the activities required to manage lameness was highlighted by farmers and advisors. Lameness management requires farmers to perform multiple actions consistently and alongside other demands on their' time. The time taken to see results could also pose challenges. This was highlighted in the context of assurance schemes. Whilst several schemes were thought of as useful and noted as driving a decrease in lameness, particularly in the dairy sector, caveats were raised. Several advisors stated that due to the risk and fear of losing contracts with dairy companies, farmers were being rewarded based on the mobility scores they provided, even though the score might be inaccurate. The time needed to make changes to see a reduction in lameness with these schemes, was therefore not always feasible.

“*Well, you reckon this is realistic?” “Well, no, it's not.” Then you say, “Well why aren't these scores realistic?” and they all say, “It's pressure. It's pressure to hit those targets.” It's just like if you go to the doctors and they say, “How much booze do you drink?” you give them what they want to hear. So, I think that can be sometimes a bit damaging when it comes to lameness, not alcohol but that pressure.”* (Advisor 11)

Resource constraints also have a bearing on opportunities to manage lameness. This included the cost of practical measures to reduce lameness incidence in the first instance, as well as the economic consequences of having lame animals. The latter included reduced productivity (lower milk yields or longer time to fatten) and the inability to take lame animals to market (due to legislation preventing the movement of lame animals and that a lame animal won't fetch as good a price).

“*A buyer isn't going to give the most amount of money for stock that is slightly lame or isn't quite right. So, the sellers know, if they do have an animal that isn't correct, those sheep or cattle aren't going to meet a decent price So there's no point bringing them.”* (Advisor 15)

Farmers' views on healthcare spend were also highlighted by farmers and advisors, particularly as to whether it was perceived as a preventative investment or a short-term cost—“*the preventative stuff, that money is spent to save them money, so it's an investment, whereas the fire brigade stuff it's all too late, isn't it*?” (Advisor 7). However, the ability to invest in some cases was restricted. For the sheep industry, it was highlighted that the profits are so small that investment is difficult.

“*Very rarely for a sheep, purely economics, it's £35 a call out or even £36 now. Probably a ten-minute consultation charge on top of that and then after you've told them what the problem is anyway and then another £20 for doing something, so you end up with a bill which exceeds the economic value of the animal. So, I don't often see the vet on here.”* (Farmer 8)

The value of spending on more preventative actions, either for veterinary input or on-farm changes, was also highlighted by several advisors. Farm advisors mentioned that, despite the economic costs, correcting lameness corrects a lot of other things too, with further benefits to the farm, and so should be seen as a worthwhile investment.

“*The reason I mention it is because for these sorts of things, if people are on a plan, in their eyes they've paid for it so that's a real boon with beef and sheep clients to getting out and about on farms, say, “Look, why don't I come down and have a look?” It doesn't cost them anything really. The amount they pay is just, all you do is if a client wanted to go from pay as you go on to a plan, you would just look at how much they've spent on time and visits over the last twelve months and divide that number by twelve… So that sort of thing helps with preventative stuff or any work really, they'll get you out sooner rather than later.”* (Advisor 10)

A lack of data on livestock was seen as problematic and limiting opportunities for management given no or poor data could create issues for identifying or managing lame animals: “*If you don't have any data then you have a problem because you don't know what you're doing*” (Farmer 14). This emphasizes the value of good record keeping as part of knowledge generation. Good record keeping was not always explicitly or systematically done by farmers, as one said; “…* I don't measure and monitor lameness, we're just reacting”* (Farmer 21).

Environmental constraints which could limit opportunities for farmers to implement best practice included the weather and the physical infrastructure of the farm. For example, one farmer did not use footbaths for treating hoof conditions due to “*Just logistics as much as anything, where we put it and all the rest”* (Farmer 19). The costs needed to update facilities was also cited as prohibitive.

“*But some farms, the financial pressure on them makes it very difficult for them to get where they want to be. They have taken on a farm that has got really old sheds that need updating and modernising, but that's a massive input for those farmers. I think we've got to look at the bigger picture.”* (Advisor 3)

Several resource constraints also affected advisors as well as farmers. For example, a lack of facilities (e.g., a crush for cattle) could limit an advisor's role. “*The big majority don't have, like, foot trimming crushes; they'll have the crush, but it's not ideal”* (Advisor 16). In addition, for the more specialist advisors, the farms described as needing their expertise the most were often those that could not afford them.

“*The farms that probably need me can never afford me because they aren't making enough money, aren't producing enough milk and aren't managing their time well so they can never afford me both from their own time point of view and the economics of their business.”* (Advisor 9)

The physical opportunity constraints experienced by farmers also limited social opportunities through a reduced engagement between advisors and their clients. Consequently, this impacted on opportunities for advisors to develop relationships with farmers and be involved in lameness management. This was particularly the case for beef and sheep farms where vets were used as needed rather than on a contract basis, as was common in the dairy sector, due to their cost. This limited call outs on farm.

“*Our vet is the main source of advice if we have a problem, we'll ring a vet up, we rarely have a vet on the farm now, you tend not to have a vet on the farm for sheep because you kind of know what your problems are and the more you use your vet for advice now…”* (Farmer 28)

Opportunities for farmer-vet interactions were further hindered in some areas by a lack of advisors with perceived appropriate specialist expertise. For advisors where there was a lack of regular contact, this also created challenges in terms of maintaining relationships and understanding a particular farm context. Some advisors had sought more proactive solutions to this such as monthly payment schemes, or linked calendar reminders for health plan follow-ups.

#### 3.2.3 Motivation

Motivation was linked to several aspects of lameness management including in relation to the gap between possessing knowledge and its application. There was often an acceptance that animals would be lame, especially for sheep.

“*I was watching a vet's meeting with farmers, and he put a picture of a lame sheep on the screen, and he said to the audience: “Describe what you see there,” and one man stood up and said: “Normality,” and everyone laughed but it probably wasn't too far from the truth because every farm that has sheep has lameness in different degrees.”* (Farmer 28)

The tolerance of and/or habituation toward lame animals across all three sectors was also raised.

“*They are working with the cows all the time, and they are just not seeing… it becomes the new normal to them. The lame cows that are just a little bit lame, they don't notice them, so they are the cows that we should be looking at, to stop them going really lame*.” (Advisor 3)

This ever-present and often chronic nature of lameness was viewed as frustrating particularly in the dairy and sheep sectors, with a realization that “*…you need to keep on top of it all the time.”* (Farmer 24) and that “*No matter how much of the pre-emptive stuff you do you're still going to get issues, you're still going to get cows go lame for whatever reason.”* (Farmer 15)

Several of the constraints identified in Section 3.2.2 were thought to not always be under the farmers' control. Together with the inevitability of lameness, and farmers' habituation to it, farmers' motivation to make change was impacted alongside their perceived capability or self-efficacy in changing behaviors. However, it was acknowledged (particularly by advisors) that advisor management can play a role here in changing mindset and overcoming de-motivation.

“*It is and it's also human nature, “I can't do anything about it. All sheep get lame sometimes,” and all the rest of it. It is just trying to change that mindset.”* (Advisor 18)

Convincing advisors and farmers they can effectively address lameness and work toward meeting lameness targets, can therefore be challenging.

Raising the issue of lame animals with farmers in the first instance can itself be difficult. The pain experienced by the animal makes it an emotive issue and a motivator to reduce incidence. Lameness can also have a stigma attached to it, and as such could be viewed as a reflection of an individual's ability and reputation as a farmer from both within and outside of the industry.

“*…a lot of farmers, in my opinion, wouldn't recognise how much lameness they've got and might find it difficult to face up to that because no one likes to feel they're being cruel. No one wants to be seen as having a lot of lame cows*.” (Advisor 14)

Given the resource constraints for some farmers, whilst good intentions/management was recognized by advisors, it was acknowledged by farmers that their current position/state was not where they desired to be. This caused frustration as well as contributing to a perceived lack of control for farmers.

### 3.3 Enablers of lameness management

Several enablers of lameness management were also identified. These were often linked to farmers and advisors. Opportunities to promote good management were created through good record keeping, and membership of assurance schemes. Social opportunity for developing and maintaining farmer-advisor relationships was viewed as important. Tied to this was the importance of capability for advisors, especially in relation to knowledge of the farm context and having good communication skills. As such advisors can play a crucial role in addressing several challenges surrounding lameness management through improving farmers' psychological capability and reflective motivation, understanding farmers' capacity for change, and focusing on farmers' motivation, such as reframing the nature of barriers. Both the good farmer and good advisor identities provided important motivators for good practice.

#### 3.3.1 Capability

For advisors, their capability and role as a “good advisor” was intrinsically linked to understanding their clients' individual farm context This helped with acknowledging any constraints clients may have, and subsequently recognizing the need to have to work with the resources and environment available and tailoring advice “*So, it's just layering on the knowledge with actually what is practical on the farm.”* (Advisor 17).

Farm advisors' communication and interpersonal skills are therefore important for helping to address several barriers. This included the perceived lack of control and inevitability of lameness perceived by farmers and helping with their ability to make manageable change. “*And building self-efficacy, you know. If you get a win in one area and set a very realistic goal, get a win, celebrate the win and that's going to build confidence for making more changes*.” (Advisor 14). As one advisor highlighted, it is about nudging them toward what to do: getting them to tell you what they feel they can do; giving the farmer ownership and accountability; and working with them. Advisors' ability to communicate and work with farmers is important within this.

The role of advisors was seen as shifting and becoming more than just about knowledge transfer. It increasingly involves the ability to be able to facilitate and empower farmers to make change through encouraging intentions, setting goals, and monitoring progress. Several of the more specialist advisors interviewed were doing this or taking steps to move toward this. This acknowledgment of a changing role was accompanied by an awareness of the changing skillset required of farm advisors, in particular vets, to best work with their clients.

The “good advisor identity” was an important motivator for advisors, including for changing their practices. Several elements important to this included staying up to date and improving confidence, developing communication skills beyond those covered in basic training and working to maintain relationships.

“*… I've also done more motivational interviewing training, facilitation training. I suppose it's stuff that I've picked up but where I thought, “That might be a benefit,” I've also done that training. So more the extra stuff I do now is not so much on learning, it's more on disseminating I suppose and about knowledge exchange.”* (Advisor 11)

“*We've done a lot of vets CPD, what is it that sheep farmers want and why do they want it? And a lot of that comes out as, actually, they [farmers] want a vet who's interested in their flock, who gives a robust reliable advice, who's independent, and understands the flock from a business point of view. So, if you talk to vets, there are a lot of vets who aren't actually confident in some of those things. And so, the vets themselves felt that they weren't able to provide that.”* (Advisor 12)

Part of this identity and revised skillset included understanding the capacity of farmers for change (both as individuals and within the farm environment) and finding ways of making information and advice relatable to farmers. This included working “together” with them, and advisors (vets) knowing when to spot a “cry for help.”

“*But I think these schemes that people are on, they are fantastic, and they do a good job to pinpoint problems, but it's trying to get the farmer to… we don't want to overload him and make him feel as though it's helpless, or that he's got to knock the whole farm down to start again. …We should be pinpointing the little things that he can do easily, with a view to changing things as we progress on.”* (Advisor 3)

Finding routes into engaging with, and having conservations with farmers was also important, including the need for advisors to be proactive in creating opportunities to do this. This regular contact helped to develop relationships, build trust and rapport.

“*We've got Google Calendars that brings up reminders for the vets to ring people at certain times of the year to get that work booked in. Reception are very good about doing vaccine reminders and making sure that that all happens. So, it's all about, you can't wait for the farmer to contact you, you've got to very push be ahead. Yeah, and drive it..*.” (Advisor 7)

#### 3.3.2 Opportunity

Farmers and advisors described how externally set lameness targets could provide a useful opportunity for positive behavior change provided they are realistic, have clear actions associated with them. Good record keeping (e.g., detailing monitoring activities, mobility scoring, individuality, growth rate etc.) was thought to be essential to good management and was often a requirement of assurance schemes linked to set lameness targets. Farmers and advisors were aware of a range of different lameness targets. This included those set nationally as part of assurance schemes (i.e., Red Tractor or RSPCA Assured), through contracts between retailers or milk suppliers, or as discussed in health plans with advisors:

“*So, therefore those schemes have driven those changes rapidly when it's been about kind of milk buyers and therefore contracts have driven those things. So, there's no doubt that's kind of changed. And that can be quite rapid.”* (Advisor 8)

The value of good, consistent advisor-farmer relationships was recognized by farmers. Many interviewees reported strong relationships with vets which created opportunities for interaction. This allowed vets to be the first port of call for advice when lameness management needed intervention beyond what the farmer could provide.

“*Yes, I personally don't like chopping and changing, and I think vets are very important that they're stable because they know the history of your problems. They can analyse… they know obviously yourself as well, that means a lot.”* (Farmer 14)

“*… you've got to trust the vet and their decision-making, and it's a costly thing using your vet but you get your money back by reassuring that actually you're only treating what needs to be treated and it's not just the animal. They're looking at the environment as well and what they're kept in and go through all sorts of things and causes of what the disease process is. So, you're getting a whole feel. They see things from other farms as well, so they'll say well, actually, we've had some cases of this locally to you, as well. So, they see the bigger picture.”* (Farmer 24)

“*I finally plucked up the courage and left and went with a new vet and proactive, just generally good and you can sit down and actually have a meeting and what you're discussing with the vet is actually listened, they listen to you and actually act upon it and vice versa. Whereas the other vets, it was just sort of lip …”* (Farmer 17)

Farmers valued ongoing relationships, and the broader experience advisors could bring, with the acknowledgment that farmers also need to be receptive to advice. The importance of developing relationships between stakeholders was also recognized by advisors, both farmer-advisor and advisor-advisor, given the opportunity for engagement this provides. The latter was particularly the case in the dairy sector (although examples elsewhere were presented), being beneficial for all parties involved. Part of this involved recognizing your own limitations and knowing when to work with others.

“*So, I can be quite honest and say I haven't trimmed a cow's foot for two or three years because we have two very good foot trimmers locally that we get on with really, really well. And if we have a lame animal, we'll potentially go out and see it, and then if we can't restrain it, we'll get the foot trimmer.”* (Advisor 7)

“*Next step's call the vet. You know, if we've got a lot of lame sheep that's out of my jurisdiction. That's the next step. That's a vet, you know. We've got a massive group of sheep. We've pulled them all out, got them isolated so they can be treated. The next step is, ask a vet to come in and swab it or look at it, give you advice on how to treat it.”* (Advisor 13)

Like farmers, advisors recognized the importance of developing relationships with their clients. Several advisors, in particular vets, highlighted the value of seeing the same vet or familiar faces; as one said of farmers, “*they just want to get through to someone who knows who they are*” (Advisor 10). This emphasizes the value of relationships, which as one advisor described, “*…if you've got a good relationship with the farmer, you can establish where the problems are, and then try your best to help them out …*” (Advisor 2). An important part of this was the need for advisors, including vets, to have good communication and interpersonal skills. This was illustrated by one advisor—“*Improving the knowledge isn't the thing really. It's all about simplifying it”* (Advisor 14*)*.

#### 3.3.3 Motivation

Several individual and cultural factors motivated lameness management as part of the “good farmer” identity, and a need to have a healthy, productive flock/herd. This was both externally driven by a need to comply with assurance schemes, and internally motivated through a desire to have a healthy herd or flock. Some interviewees also perceived a social pressure to have low or no lameness via external (assurance/legislation) mechanisms, as well as from other farmers in the industry.

Low levels of lameness were linked to pride, with lame animals thought to reflect badly on a farmer's reputation and capability. Farmers thus wanted to be seen to have healthy animals “*…. But I also think it's a pride thing is, ‘We haven't got a lot of lame sheep.”'* (Advisor 13).

It was acknowledged that given the visible physical presentation of the condition, lameness was a particularly important cue to an animals' health and welfare status even for those with little knowledge of farming, such as the public. As such, having lame animals can easily seen, and could be taken as a reflection on individuals' stockmanship given the welfare implications.

“*… everybody can see a lame sheep and it probably is something that bothers the general public more and we probably need to be more aware of it…”* (Farmer 28)

Being viewed as a good farmer identity was a motivator for good practice. To be regarded as a good farmer, with a low incidence of lameness, several different qualities and practices (linked to individuals' capability) were needed. These included being proactive, having good records to monitor lameness, having the right knowledge and skills for lameness identification and management, and working with advisors where needed. Likewise, being a good advisor was a motivator for advisors behavior. To be considered as a good advisor several skills were required of individuals, linked to their capability.

## 4 Discussion

### 4.1 Overview

In line with previous research, the findings reported here highlight that lameness is viewed as complex and multifactorial, and that a range of factors influence farmer and advisor lameness management behaviors. The use of the COM-B model, specifically the framework of capability, opportunity and motivation (55), has provided a novel, useful means of conceptualizing these factors and provided an understanding of some of the factors that may influence particular behaviors (57). Whilst other approaches to qualitative analysis, e.g., thematic analysis or grounded theory, all offer valuable means of identifying factors that influence lameness management practices, the COM-B framework has provided a specific means of categorizing factors in relation to specific behavioral constructs. It considers how these are interlinked and in turn can influence behavior. Given the link between the COM-B model and the broader BCW, this categorization also facilitates consideration of the most suitable interventions based on the behavioral constructs identified.

The inclusion of both farmer and farm advisor perspectives provides new insights on lameness management and highlights the importance of understanding multiple stakeholder perspectives and their interactions in relation to this complex health and welfare issue. What emerges from the analysis are three aspects linked to behavior which could enable more effective lameness management. These are, removing physical and social barriers to opportunities for lameness management; improving psychological capability and motivation for lameness management; facilitating relationships and developing communication between farmers and advisors. Each of these is discussed in turn.

### 4.2 Removing physical and social barriers

Several barriers to enacting recommended lameness management practices were identified, with physical opportunity, rather than physical capability, often a limiting factor, both for farmers and advisors. For farmers this was often in relation to resource constraints, including time, the farm environment and finances, as noted elsewhere (41, 45). Advisors acknowledged these and the effect they were likely to have, and they also realized these constraints could affect their role including the affordability of more specialist advisors for farmers who would benefit from their expertise most.

Time was also a barrier, both in relation to farmers' time to enact measures on farm (investment time) but also the time needed to see any changes take effect (payback time). Considering payback time is important if measures are in place and are being monitored. Suitable time needs to be allocated for changes to take effect without penalty or risk. This is particularly the case for ensuring the sustainability of changes and their impacts in relation to targets associated with assurance schemes, contracts, and farm subsidy payments.

The farm environment could also pose challenges. Farmers need to have the physical and economic resources necessary to carry out the management response in question (74), as do advisors. For example, having access to appropriate facilities to ensure safe animal handling for both themselves and livestock. Farm facilities are a notable barrier to lameness treatment (41), with the capital costs associated with changes to physical infrastructure usually requiring direct financial incentives (35) to support changes. Economic considerations are also important in relation to lameness (27), with research indicating that farmers can feel financially constrained in their efforts to improve matters on farm (2). Economic considerations were raised at several points across the interviews for both farmers and advisors. As well as support for capital investment, studies have shown improved market prices, e.g., an increase in milk price, as providing more economic flexibility for investment on farm (2). This raises the question of how farmers can finance changes if the market does not support them to do so and highlights a potential role for government intervention.

Advisors highlighted that several factors were linked to the level of perceived control farmers had in managing lameness. The link between (perceived) resource constraints, level of perceived control and psychological capacity create a vicious cycle of lameness management for some farmers. Interventions aimed at breaking this are therefore critical. Farmer–advisor relationships are likely to be central to this, particularly farmer-vet relationships, given the acknowledgment of their changing role, in relation to influencing farmer behavior (79).

### 4.3 Improving capability and motivation

The psychological burden of lameness management emerged as an important area to consider. Findings from the interviews indicated that whilst knowledge surrounding lameness would appear to be good, habit, the chronic nature of lameness, its emotive nature, and the perceived inevitability that animals will become lame can all contribute to deficits in lameness management. An individual's perceived behavioral control has been found to be influential in farmer behavior (35), with dairy lameness management in particular described as an “endless burden” (42). The findings presented here reflect a need for the recognition this can play in relation to an individual's motivation and capacity for lameness management. Previous research has indicated reasons farmers may not act on advice including factors such as low self-confidence, habit and a desire to maintain simplicity (51). This is particularly pertinent when considering future intervention strategies and how best to enable farmers to make changes when they view lameness as inevitable and outside of their control. Farm advisors could play a crucial role in helping to shift farmers' perceptions toward seeing how lameness can be feasibly and realistically managed.

The “good farmer” identity was also an identified motivator of behavior. Within this there are elements associated with pride in a healthy herd/flock as well as empathy and a desire for a good public image (2). This more intrinsic motivation has been shown to be important in other elements of farming such as adopting conservation practices (47). Lameness is a particularly visible condition, even to those with limited knowledge about the farming industry and animal health. Both farmers and advisors reflected on public perceptions is during the interviews. Reducing lameness is therefore important for helping to maintain this identity.

Whilst farmers in this research could clearly describe lameness, and lameness management and prevention techniques, advisors described how the application of knowledge was not always effective in practice. This was due to perceived barriers or constraints as well as a habituation or entrenched prior beliefs leading to the involuntarily underestimation of lameness in flocks and herds. Accurate assessment of lameness prevalence is important for ensuring appropriate prevention measures and prompt treatment (14). Studies have shown that farmers can have a low awareness or knowledge of lameness, underestimate lameness in their herd or flock, or becoming desensitized to lame animals (14, 37, 39, 40). Previous research has shown that the emotional aspects of motivation affect behavior (13) and that habit is often a barrier to changing farmer behavior (80). Research has shown that changing practices can be difficult (81). The findings reported here suggest that regular opportunities for reviewing lameness could be beneficial. This could be through external assessors, joint lameness scoring with an advisor, improved opportunities for training, and the incorporation and encouragement of good record keeping.

Having opportunities to share good practice and effective knowledge transfer for disease control is therefore important (74). Given the reluctance demonstrated by some farmers to change practices the trialing of new management practices on a small number of animals has been discovered to help farmers adopt new practices. This is through enabling them to try out new processes in a risk-reduced, and potentially easier to implement, manner (31), and establishing the value and ease of adopting behaviors (35). Peer-to-peer learning has also been identified as a promising means of helping farmers to change behavior (82). Facilitating knowledge exchange within and between different groups is important, and provides a good way to acknowledge differences between farms (44) thus helping to address farm-specific factors.

### 4.4 Facilitating relationships and improving communications

Communication and social opportunity are important factors within lameness management, including the role of farmer-advisor relationships, and in particular the relationships between farmers and vets. Previous research has highlighted the importance of communication (35), good interpersonal skills (57) as well as social opportunity for farmers and vets in animal health management (55), and that farmers value the relationship with their vet (32, 83). For example, specialist veterinarians are a preferred source of lameness information for sheep farmers (84), with farmers placing a high value on personally relevant advice (56). Research by Prosser et al. (74) discovered that farmers who were close to their vet were more likely to trust them and more likely to undertake measures to prevent and control disease, in their case BVD, with farmers more receptive to advice when it is delivered through a process of mutual respect [(85) in (56)]. Developing these relationships is therefore important, especially within the beef and sheep sectors where there is typically less engagement with the vet on beef (41) or sheep farms (45). Overcoming economic challenges, such as the cost and affordability of veterinary services within this is important.

The nature of the communications needed to facilitate such relationships may require different training for those who work with farmers, going beyond just knowledge transfer. Communication is an important component of veterinary education (86). Research has suggested vets need to further develop their communication skills (51). These skills are seen as an important part of the good advisor identity. Veterinary medicine has tended to focus more on the technical content of messages (49) with vets adopting a more paternalistic style of communication (87). This could hamper relationships as farmers often report that they prefer to be treated as equals (53) [(85) in (56)]. Using different communication strategies can be effective in changing farmer behavior (49) including reaching more traditionally hard-to-reach-farmers (88). This could include ensuring a range of different media are used, as well as tailoring or customizing messages based on farmer motivations (88). Research has also indicated that better skills in understanding and influencing behavior would be useful for vets adapting to a more preventative medicine-based role (79). Developing advisor communication skills, to help facilitate farmer behavior change, improve farmer self-efficacy and a more proactive health management role would be beneficial. This aligns with a broader recognition of the changing role of the veterinary profession (79).

It is also acknowledged that industry collaboration is likely to be important in preventing lameness (41). This research renews calls for improved collaboration and communication between farmers and their advisors, as well as additional support from industry in encouraging and sustaining positive change. Wynands et al. (42) describe this as a shared responsibility between different stakeholders. Industry-led initiatives have been rated very highly as strategies for improving health and welfare (15). Market or compliance-based rewards can therefore be useful if these are clear, and the reward obvious (35). Whilst thought to be useful by participants there were caveats to their implementation such as pressures leading to inaccurate and underreporting of lameness levels. Sufficient time for change and the setting of realistic targets to drive improvement, whilst ensuring that those who may be struggling are supported, are important considerations within this.

### 4.5 Recommendations, limitations, and future research

The factors identified above, including the interaction between the identified elements of the COM-B model, provides the rationale for multiple, targeted interventions aimed at both farmers and their advisors. This also aligns with previous research which has highlighted that applying a range of approaches is more effective in changing behavior [(89) in (28)] in relation to lameness control (2). Indeed, the range of factors identified here supports the need for internal and external motivators (i.e., more enabling factors or more incentivization based interventions) to reflect this (47).

Once an understanding of behavior is established, the BCW next recommends the identification of appropriate intervention functions (see Figure 1 for an overview) (1). Based on the above results, enablement can be considered as a prime means to target behavior change. Enablement is “increasing means/ reducing barriers to increase capability (beyond education and training) or opportunity (beyond environmental restructuring)” (1). In the context of lameness, enablement strategies could correspond to addressing several of the physical and social opportunity and reflexive motivation-based factors identified in the research. Involvement and co-ordination with advisors, and developing advisor communication skills will be central to the delivery of these strategies. Given the importance of farm context, management of the situation these should be flexible (20). This will enable the development of preventative solutions that tie in with farming routines (90), and reflect the predominant causes of the lameness in individual herds and flocks.

In addition, research has shown that close communication, e.g., one-to-one or in a group, may well be more effective or motivating for farmers in managing lameness (91). This requires opportunities for farmer-advisor interactions to occur and that advisors are trained to facilitate these discussions. Herd/flock health plans present one of the few opportunities to provide systematic, individual, advice on an issue (79), with record keeping and good data an important part of this. However, this is usually done on an annual rather than ongoing basis, such as reviews after repeat lameness events. Access to experienced veterinary professionals is important (15), yet the (then) Farm Animal Welfare Council (92) highlighted that there is a shortage of specialist vets, particularly for sheep (93). Looking to address this skills gap is important, including the training of vets to deploy the kind of approach advocated here. More regular interactions between farmers and vets should also be encouraged, and financial support could aid with this for some farmers.

Quality assurance schemes such as Red Tractor Assurance (94) or industry schemes such as those run by milk processors are likely to be using incentivization and coercion to encourage change and meet specific lameness targets. Solely using compulsory means to drive behavioral change will typically mean these changes will only last as long as the coercion or enforcement exists (27). Additional, voluntary behavioral change interventions, based on either internal or external motivation, are therefore important (27). These should align with actions and interventions taken by other stakeholders to ensure a more co-ordinated approach and ensure reasonable time periods to make change.

Whilst this research provides several practical suggestions, it should be noted that different farmers will have different motivations. This was acknowledged by several of the advisors in the research who talked about the importance of knowing individual client motivations and contexts. This is acknowledged in the literature (50) particularly around lameness best practice and non-compliance behaviors (13, 48). Given the qualitative nature of this research farmers were not segmented or categorized based on the COM-B elements. Future research should explore this in the context of lameness management. This could include looking to establish preferences for intervention types including which mechanisms would best enable behavior change for the barriers identified in relation to reflexive motivation, physical and social opportunity, and psychological capability. Subsequent studies could then look to test the identified behavior change interventions with farmers. Research should also look to explore how advisors can further develop their communication skills alongside ongoing professional practice. Whilst knowledge of lameness was identified as good amongst farmer participants it should be noted that participants volunteered to take part and therefore may not be a fully representative sample. Future work could look to explore the COM-B elements with a broader sample of farmers, and in relation to other animal health and welfare issues.

## 5 Conclusion

This research explored barriers and enablers to lameness management on UK cattle and sheep farms using a behavior change framework. The research used the COM-B model as a means of better understanding the mechanisms underlying lameness-related behaviors. This enabled an exploration of how farmers' and farm advisors' capability, opportunity, and motivation impact lameness management. The research highlights the value of utilizing such frameworks within animal health and welfare management, and for thinking about how the barriers and enablers of management behaviors are conceptualized and interlinked, to aid the identification of appropriate interventions. The inclusion of both farmer and farm advisor perspectives provides new insights on lameness management and highlights the importance of understanding multiple stakeholder perspectives and relationships in developing effective responses.

## Data availability statement

The datasets presented in this study can be found in online repositories. The interview transcripts for this study will be available in the UK Data Archive following an embargo period. For questions about the data availability please contact the corresponding author.

## Ethics statement

The studies involving humans were approved by Newcastle University Faculty of Science, Agriculture and Engineering Ethics Committee. The studies were conducted in accordance with the local legislation and institutional requirements. The participants provided their written informed consent to participate in this study.

## Author contributions

BC: Conceptualization, Data curation, Formal analysis, Investigation, Methodology, Writing—original draft, Writing—review & editing. AP: Data curation, Funding acquisition, Methodology, Writing—original draft, Writing—review & editing. NM: Data curation, Formal analysis, Methodology, Writing—original draft, Writing—review & editing. LH: Data curation, Formal analysis, Funding acquisition, Methodology, Writing—original draft, Writing—review & editing.

## Footnotes

^1^The “Ring of Confidence” model places the farmer at the center with concentric rings of trust around them. This includes an inner ring of advisors offering trusted, credible advice and an outer ring of advisors who sit outside the management team that focus on policy or legislative developments (55).

^2^Defined as “state of mind that influences how people think about and then enact their goal-directed activities in ways that may systematically promote or interfere with optimal functioning” according to the American Psychological Association (https://dictionary.apa.org/mind-set).
