# Supplementary material for: Exploring farmer and advisor lameness management behaviors using the COM-B model of behavior change
Source: Front Vet Sci. 2024 Jan 17;11:1258906. doi: 10.3389/fvets.2024.1258906 (PMC10827996; doi:10.3389/fvets.2024.1258906)
Supplement: Supplementary file 1 [file Data_Sheet_1.PDF]

Interview no. ________________

Pre-interview questions

About you

1. What is your role on the farm?

2. Who else works on the farm/helps out on the farm?

3. Which age group do you fall into? (Please ✓)

19 years old or under	
20 – 29 years old	
30 – 39 years old	
40 -49 years old	
50 -59 years old	
60 -69 years old	
70 -79 years old	
80 plus years old	

4. How long have you been farming?

About your farm

5. a. Approximately what is the size of the farm (Please ✓)
   b. Approximately what is the amount of grazing land on the farm? (Please ✓)

	a. Farm size	b. Amount of grazing land
0.1 to 5 ha (0.1 – 12.3 ac)		
5.1 to 20 ha (12.4 – 49.4 ac)		
20.1 to 50 ha (49.4 – 123.6 ac)		
50.1 to 100 ha (123.7 – 247.1 ac)		
100.1 to 150 ha (247.2 – 370.7 ac)		
150.1 to 200 ha (370.8 – 494.2 ac)		
200.1 ha plus (494.3 ac plus)		

6. Please describe the tenure of your farm, e.g., Owner-occupier, tenant (Agricultural tenancy, Farm Business Tenancy, other type of formal or informal tenancy etc.)

7. a. Please tick all the farming activities that apply to your business (Please ✓ all that apply)

Beef suckler	
Lambing flock	
Dairy cows	
Non-dairy goats	
Poultry (chickens, ducks, geese)	
Arable/forestry	
Calf rearer	
Pigs	
Other	

Lamb store/finisher	
Dairy ewes	
Deer	
Turkeys	
Beef grower/finisher	
Ram breeder	
Dairy goats	
Game birds	

7. b. If other, please specify all other enterprises	

8. What is/are your County Parish Holding Number(s)?

County Parish Holding number(s)	

About your animals

9. Approximately how many animals of each type do you currently keep on your farm?

	Approximate number of livestock							
	0 – 9	10 – 49	50 – 99	100 -149	150 – 199	200 – 249	250 – 299	300 plus
Beef (breeding)								
Beef (non-breeding)								
Dairy (breeding)								
Dairy (non-breeding, e.g., followers, youngstock)								
Sheep (breeding)								
Sheep (non-breeding)								

10. What are the main breeds of cattle and/or sheep on your farm? (list all breeds mentioned)

Beef	
Dairy	
Sheep	

11. Do you keep a pedigree and/or a commercial herd/flock? (Please ✓ all that apply)

Livestock type	Pedigree	Commercial	Purebred
Beef			
Dairy			
Sheep			

12. Are you part of any accreditation or assurance schemes (e.g., Red Tractor, RSPCA Assured, Arlagården etc.)? If so, which one(s)?

Interview questions

Background on the individual and the farm

1. [using information from the pre-survey] – You told us your role on the farm was …… What does this role involve?
   How has the current Corona virus/Covid-19 shut down impacted your role?
   (Were you always in this role, or have you had other roles on this (or other) farm(s)?)

2. [Using information from the pre-survey] – You told us …… works/helps out on the farm, and what do they do?
   How has the current Corona virus/Covid-19 shut down impacted this?
   (Paid versus unpaid, family versus non-family, full-time versus part-time and casual, times of year, specific roles and responsibilities, how 'hands on' are they?)

3. We would like to have a better understanding of your farm and the environment you work in, could you describe the layout of your farm for me.
   Prompt the interviewee to include the following features,
   - Where the farmhouse is in relation to the other agricultural buildings,
   - Locations of farm buildings in relation to each other and other farm infrastructure,
   - Location of fields (names of fields if possible)
   - Types of tracks and locations relative to other features, e.g., entry points, biosecurity features.
   - Boundary lines and types of boundaries.
   - The topography of the land and soil types in different fields

(Other features, e.g., biosecurity infrastructure, roads, tracks, entry points, boundary lines, types of boundaries, soil types, topography of the farm)

4. [using information from the pre-survey] – You told us your most important breed(s) was/were XXX …

   a. Why do you keep these breeds?
   (What are the qualities of these breeds that you value? Is disease resistance/resilience important relative to other qualities? Is the breed important to you? Have you always kept these breeds? Why? Pedigree versus commercial animals)

b. Where do you go for information about different breeds of livestock?

(What kinds of information do you seek out? Whom do you ask? Why these individuals or organisations? Are there any breeds that you are currently interested in? Why?)

5. a. How do you make decisions about the breeding strategy(s) used?

   b. Where do you purchase animals?

   How has the current Corona virus/Covid-19 shut down impacted this?

   (Who makes these decisions? Does anyone else have a say?)

6. Throughout this interview we are interested in your history and experiences with farming. This will aid in our understanding of how things have changed over time, as well as support the work of the historians involved in the FIELD project.

   a. Have you undertaken any training? Do you have any agricultural qualifications?

   b. When did you start farming here? Did your family farm here before you? (Your history on this farm)

      (How long have your family been farming? This farm or other farms? Have you always undertaken the same activities? Any major changes to the infrastructure, livestock types and breeds on the farm?)

   c. What is your approach to farming? What are the key influences on this approach?

      E.g., education, disease events, new farms/land, changes in enterprise, technologies, biosecurity etc.

      How has the current Corona virus/Covid-19 shut down impacted this?

Disease questions

BVD (elicit any differences between beef and dairy cattle)

7. We are interested in how you think about BVD. Could you describe the disease?

   (Has your understanding of BVD changed over time? Has the way you refer to BVD changed over time? When did you first hear of BVD?)

8. What are the symptoms of BVD?

(How do you know when an animal has BVD (PI/TI)? How do you know when an animal doesn't have BVD, e.g., when buying livestock at a mart? – What the animal looks like, how it acts, other sensory cues?)

9. a. What experiences have you had with BVD? (On current or previous farms, on neighbouring farms, in the news, etc.)
(Do you have (or ever had) BVD on farm – how many animals have/had BVD (PI or TI)? If you have experienced BVD, what made you first suspect it, what did you do? What was the impact, e.g., productivity, vets fees, loss of animals and loss of trade, cost of taking actions? Have you heard about it from other sources, e.g., vets, the agricultural press, other advisors, etc.?)

b. If you have never had BVD on your farm, how do you know this?

(If interviewee has never had BVD on their current or previous farm please use the questions in the box on page 7)

10. Do you test for BVD?
(Why/why not? When did you start testing? What did you do before this?)

    a. If yes, what were the results of the most recent test? How do you usually test for BVD?
    (What method of testing do you employ? Why this method and not others? Who does the testing? How often? Do you test at a specific time of year? How many animals do you test each time? Where do you conduct the testing?)

    b. How reliable/accurate do you think the current tests for BVD are?

11. Do you vaccinate against BVD?
(Why? When did you start doing this? What did you do before this?)

    a. If yes, how do you vaccinate against BVD?
    (Which vaccine do you use? Why this vaccine and not others? Who vaccinates the animals? How often? Do you vaccinate a specific time of year? Do you vaccinate all the animals, or a sub-set? Where do you conduct vaccinations?)

    b. How reliable/accurate do you think the current vaccines against BVD are?

12. Where are the areas of risk in relation to BVD in the farm?

    a. How do you prevent BVD from entering your farm?

(practices that reduce or increase risk of BVD….)

(Areas of the farm in which animals have the greatest risk of catching BVD? E.g., where different management groups, or neighbouring herds might have contact, when taking animals to shows, trade. Practices to control BVD and relative successes of these. What else could you do but don't? Why don't you do these things? Closed herd?)

13. What do you think is the risk of BVD to your farm? (High, medium, low. Why?)

    (The chance of an outbreak of BVD on-farm)

14. What do you think the impacts of a BVD outbreak would be on your farm?

    How has the current Corona virus/Covid-19 shut down impacted this, e.g., ability to test and vaccinate, biosecurity considerations, etc.?

    (Financial, reputational, welfare, emotional etc. Approximate quantification in terms of productivity, vets fees, loss of animals and loss of trade, cost of taking actions.)

15. What is your experience of BVD eradication/management schemes?

    (Which schemes have you heard of? How do you hear about them? Have you taken part in any of these? Why/why not? What's your opinion of these schemes? Role of government policy?)

16. Where do you go/with whom do you consult for information on BVD?

If the interviewee has never had BVD on their farm

18. How do you prevent BVD from entering your farm?

(Areas of the farm in which animals have the greatest risk of catching BVD? E.g., where different management groups, or neighbouring herds might have contact, when taking animals to shows, trade. Practices to control BVD and relative successes of these. What else could you do but don't? Why don't you do these things? Closed herd?)

19. a. Do you test for BVD?

    (Why/why not? When did you start testing? What did you do before this?)

    b. How reliable/accurate do you think the current tests for BVD are?

20. a. Do you vaccinate against BVD?

    (Why/why not? When did you start testing? What did you do before this?)

    b. How reliable do you think the current vaccines against BVD are?

21. A. What do you think is the risk of BVD to your farm? (High, medium, low. Why?)

(The chance of an outbreak of BVD on-farm)

22. What do you think the impact of a BVD outbreak would be on your farm?

How has the current Corona virus/Covid-19 shut down impacted this, e.g., ability to test and vaccinate, biosecurity considerations, etc.?

(Financial, reputational, welfare, emotional etc. Approximate quantification in terms of productivity, vets fees, loss of animals and loss of trade, cost of taking actions.)

23. What is your experience of BVD eradication/management schemes?

(Which schemes have you heard of? How do you hear about them? Have you taken part in any of these? Why/why not? What's your opinion of these schemes? Role of government policy?)

24. Where do you go/with whom do you consult for information on BVD?

25. a. How much of a problem is BVD relative to other diseases on the farm?

(E.g., Johnes, bTB, lameness etc. How much of a concern is it now compared to 5 or 10 years ago?)

b. How much of a problem is BVD relative to other issues affecting the farm?

(E.g., Brexit, changing consumer tastes, tenancy and succession issues, the current Corona virus/Covid-19 pandemic, etc.)

Lameness (elicit any differences between beef and dairy cattle and sheep)

26. We are interested in how you think about lameness. Could you tell us how you would describe lameness?

    (What conditions and injuries would you consider to be 'lameness'? Elicit differences between beef, dairy cattle and sheep)

27. What are the symptoms of lameness?

    (Elicit sensory cues, and any differences between beef, dairy cattle and sheep)

28. What experiences have you had with lameness? (On current or previous farms, on neighbouring farms, in the news, etc.)

(Approximately how many animals are currently lame? Are there seasonal/ between year differences? What kinds of lameness do you experience on your farm – infectious, non-infectious? Differences between beef and dairy cattle and sheep? Approximate quantification of impact, e.g., on productivity, vets fees, loss of animals, loss of trade, cost of taking actions)

29. What causes lameness on your farm?
    (Elicit any differences between beef and dairy cattle and sheep)

30. What are your aims/goals/management strategy for lameness on your farm?
    How has the current Corona virus/Covid-19 shut down impacted this?
    (Has this changed over time? How feasible is eradication as a goal? Elicit any differences between beef and dairy cattle and sheep)

31. How do you monitor and test for lameness?
    (On-going monitoring, e.g., lameness scoring systems, or testing for infectious lameness)

32. How would you manage/treat an individual lame animal?
    (Elicit differences between beef, dairy cattle and sheep. When would you consider culling? Are there any measures you would take to protect the rest of the herd?)

33. a. Where are the areas of risk in relation to lameness in the farm?
    (discuss the insides of buildings – seasonality and weather are important here)

    b. How do you try to prevent lameness on your farm? (discuss the insides of buildings – seasonality and weather are important here)
    How has the current Corona virus/Covid-19 shut down impacted this?
    (Practices to control lameness and relative successes of these. Vaccination? Foot trimming? What else could you do but don't? Why don't you do these things?)

34. What is your experience of lameness management schemes?
    (Which schemes have you heard of? How do you hear about them? Have you taken part in any of these? Why/why not? What's your opinion of these schemes?)

35. Where do you go/with whom do you consult for information on lameness?

36. a. How much of a concern are BVD and lameness relative to other diseases on the farm and other issues affecting farming?

(E.g., Johnes, bTB etc. How much of a concern is it now compared to 5 or 10 years ago?)

b. How much of a concern are BVD and lameness relative to other issues affecting the farm? (E.g., Brexit, changing consumer tastes, tenancy and succession issues, the current Corona virus/Covid-19 pandemic, etc.)

Knowledge networks

37. We are interested in the connections between your farm and other parts of the farming sector. Could you describe where,
    a. Your nearest neighbours are,
    b. Where the marts, vets and abattoir that you use are,
    c. Where the nearest settlements are? Disease status of neighbouring farms,
    d. Nearest agricultural show or where you show your animals.

38. Where do you go/with whom do you consult for information on livestock health, welfare and disease?
    (The sources of information and the modes of delivery. Why these sources? Frequency of use? Do you trust them equally?)

39. When you started farming what were the main sources of information? How have these changed over time? (Use of the timeline to explore change and continuity in relation to sources of information)
    How has the current Corona virus/Covid-19 shut down impacted this?
    (What causes changes? Gradual or dramatic changes? Trust in the different sources. Have these changes made the interviewees more reflective, or challenged their preconceptions? Friends and family, neighbours, the agricultural press, the internet, farmer groups [which?] vets, other advisors [who?] Which are the most important? Has it always been this way?)

40. [using information from the pre-survey] – You told us you are a member of …… scheme, why did you join this scheme?
    (When did you join? How did you first find out about the scheme(s)? What are the pros and cons of the scheme? Are there any that you have left? Are there any you are thinking of joining?)

    b. What are the requirements of the schemes you have joined?

c. Do you get guidance on how to follow the rules of the schemes you have joined?

(What types of guidance do you receive? What types of guidance do you seek out? What format does this guidance come in?)

d. Are a member of any herd health schemes? How useful to do find the scheme?

41. a. Do you share your knowledge and experiences with others (E.g., on-farm experimentation, training, use of social media etc.)

    (Formal and informal methods)

    b. What do you do? (focus on those with livestock health and welfare aims)

    How has the current Corona virus/Covid-19 shut down impacted this?

    (What kinds of knowledge do you share? In what forms? Who do you think is your audience? How often do you share/produce knowledge? What kind of response do you get from this?)

42. Are you a member of any farmers groups or organisations? Which ones? What do they do? (E.g., local informal discussion groups, membership of AHDB, NSA etc.)

How has the current Corona virus/Covid-19 shut down impacted this?

(Focus on those with livestock health and welfare aims. What do you benefit/gain from your involvement?)

"Philosophy of farming"

43. What is a good farmer?

(What personal qualities make a good farmer? What does a good farmer do (processes/activities)? Do you think other farmers would agree? Do you think the general public would agree?)

    a. How do you know when someone is a good farmer?

       (The outputs and outcomes of being a good farmer?)

44. Are livestock health and welfare the same thing, or are they different? Why?

45. Who is responsible for livestock health and welfare in the UK? Why? What should their responsibilities be? (Do all those responsible have the same responsibilities?)

46. Is there anything further you would like to add?

Interview no.	

Advisor interview questions

Pre-interview questions

1. What is your current role?

2. How long have you been in this role?
(At this practice/organisation? At other practices/organisations?)

3. Tell us about your veterinary practice/organisation/company/business.

a. Number of people employed (including interviewee)	
b. Percentage split of clients (companion/equine/farm animal)	
d. Your furthest client (approx. distance in miles)	

4. a. Do you have a specialism? What is your specialism?
(Do you have RCVS accredited specialist status? E.g., small/large animals, specific animal types?)

b. What farm types and husbandry systems do you work with?
(Large/small, upland/lowland, breeding/fattening, intensive/extensive, indoor/outdoor, closed/open?)

c. How much time is devoted to which species?

5. a. Are you currently carrying out work for an assurance scheme? What is your role in this respect? Which ones?
(E.g., Red Tractor, RSPCA assured, Arlagarten, Soil Association, Premium Cattle Health Scheme.)

Assurance scheme	Role and responsibilities

b. Does your practice/organisation/company run any of its own health and welfare schemes?

6. a. Are you involved in any other professional activities/responsibilities, e.g., research, policy, as part of a professional body?

b. Do any of these activities relate specifically to BVD and lameness?

?

You and your role

7. You stated that your role is … [xxx] … What does this role involve?
   What health issues do you spend most of your time dealing with? How much time is spent on routine visits v's firefighting work, in which species? How has this changed? Has your role changed at all over the past few months with the Covid-19 crisis?)

8. You said you were involved in …{xxx]… herd/flock health plans.

   a. Could you briefly outline how these are created and work in practice

   b. Do you think the herd/flock health plans change farmer behaviour in the right direction? Why?

   c. Do you think herd/flock health plans are good at improving animal health and welfare?

   d. What do you consider are the major disadvantages / advantages of herd/flock health plans?

9. You said you were involved in … [xxx] … assurance schemes.

   a. Do you think the assurance schemes change farmer behaviour in the right direction? Why?

   b. Do you think assurance schemes are good at improving animal health?

   c. What do you consider are the major disadvantages / advantages of the assurance schemes?

   d. What are the main differences between herd/flock health plans and assurance schemes?

Your relationship with farmers and animals

10. Has working with farmers changed over time? In what ways?
    (Establishing relationships, building confidence, developing trust, bedside manner)

11. Has working with livestock changed over time? In what ways?
    (Changing breeds, new technologies)

12. a. What makes a good [advisor type]/farmer relationship?
    (Relate to health and welfare outcomes; understanding the wider farm business, farmer concerns etc.)

Questions about BVD

13. What is BVD?
    (How do you talk about BVD with your clients?)

14. a. What set of events or circumstances might make you suspect the presence of BVD on a farm?
    (What other diseases or conditions might BVD appear in combination with? Elicit sensory cues. How it presents in different types of cattle, breeding versus non-breeding animals, dairy cattle, beef cattle, etc., different years and times of year).

    b. What would you do after you suspect the presence of BVD on a farm?

(Are there any standard operating procedures in your business? How do you know that you have been successful?)

c. How often do you encounter these events/circumstances?
(Have practices relating to BVD [e.g., vaccination versus BVD-free herds], ideas about BVD and responses to BVD changed over time?)

15. How much of a problem/ important is BVD is relative to other livestock diseases?
(Generally, in England, and, in their "patch", to their clients)

16. What activities on farms increase risk in relation to BVD?
(Also elicit times of year that increase risk. Is there anything you wish your clients would do differently to reduce the incidence/ risk of BVD?)

17. What do you advise farmers to do to manage BVD?
(Do you advise clients to test, vaccinate? Which methods? What are your opinions on these methods? Do you give advice on biosecurity and trading practices? Do clients take your advice? What are the challenges of delivering advice around BVD to clients?)

18. How reliable do you think the current tests and vaccines for BVD are?
(Prompt different tests – individual tissue tests, e.g., ear tag plugs, herd tests – milk, blood etc. Prompt different vaccines – single or multiple dose vaccines, use of Rispoval 4)

19. Is there anything you can do when the actions of one client negatively impacts the livelihood of another client? E.g., if you knew the BVD status of one herd was going to impact on neighbouring herds?

20. What is your experience of BVD eradication and management schemes? (Awareness and opinion of schemes in other countries and the devolved nations, role in design/delivery)

21. What should be the aim of BVD management schemes?
(E.g. reduction, eradication, responsive treatment? Scale of action – geographic and temporal?)

22. Why do you think BVD persists in England?
(Opinions of the situation in the other devolved nations?)

Questions about lameness

23. What is lameness?
(How do you talk about lameness with your clients?)

24. a. When presented with a lame animal, how do you go about determining what the problem is/what's causing it'?
(Elicit differences - e.g., sheep v's cattle, breeding v's non-breeding animals, dairy cattle v's beef cattle, different times of year, between years. Is this always done with the animal present? Chronic vs acute lameness, individual animal vs herd/flock, breed differences)

b. What would you do next?

(Are there any standard operating procedures in your business? How do you know that you have been successful?)

c. How often do you encounter these events/circumstances?
(Have practices relating to lameness [e.g., prevention, foot trimming], ideas about, response to and causes of lameness changed over time?)

25. How much of a problem/ important is lameness relative to other livestock conditions/diseases?
(Generally, in England, and, in their "patch", to their clients)

26. What do your clients do to manage different forms of lameness on their farms?
(What are your opinions of these practices? Proactive versus reactive. Vaccination against foot rot. Elicit different species, animal types. Do other advisors get involved? How is this mediated? How do you know if farmers are following your advice correctly? What do you advice farmers do? What are the challenges of delivering this advice? What do you wish your clients would do differently to reduce the incidence of lameness? Do the resources farmers have at hand make it more challenging to follow advice e.g. cattle crush, manpower, space. Does this affect your relationship with your clients?)

27. Where do you think the areas of risk for lameness are on a farm? What activities/factors increase risk in relation to lameness?

28. What should be the aim of lameness management plan/scheme?
(E.g. reduction, eradication, responsive treatment? Scale of action – geographic and temporal? Who is responsible?)

29. Why do you think lameness persists in England?
(Compare to the response of the other devolved nations.)

30. Who or what is driving the push to manage/eradicate BVD and lameness in England?
(Government, industry, suppliers, the public, etc.?)

Information and knowledge networks

31. Where do you get your information/with whom do you consult for information on BVD/lameness?
(Is staying up to date a challenge? Do you learn from peers, veterinary scientists, farmers, others who work with animals? How much of this knowledge is based on field-generated experience or research and how much on other sources? Which sources are most trusted and useful? How does this information change your practices? In what ways?)

32. Do you work alongside other professionals?
(Who do you work with? How is this coordinated? What helps or hinders you working together? What are the advantages and disadvantages?)

33. a. In what ways do you deliver advice on a group level?
(Do you run events, discussion groups, training etc? Is this a group of farmers or a group of workers on one farm? To what extent does money influence the relationship? How do farmers value free versus paid for advice? Do you think that farmers ever feel that paying for advice reflects badly on their own knowledge/skills?)

b. Do you tailor advice depending on the client
(In what ways do they tend to do this)

The philosophy of farming

34. What makes a good farmer?

35. a. What makes a good … [advisor type]…?
(Key skills/qualifications? Earning respect, establishing authority, building status, learning how to communicate with farmers? What things might your training not have prepared you for?)

36. a. Are livestock health and welfare the same thing or are they different? Why?

    b. Where do BVD and lameness fit into this, are they a health issue, a welfare issue, both, neither? Why?
    (Are they diseases or conditions? Why?)

37. Who is responsible for livestock health and welfare in the England? Why?

38. With the introduction of a new Agriculture Bill (animal health pathway) and the focus shifting to *public money for public goods.* Have you given any thought to what the implications might be for your role and the functions you might perform?

39. How has Covid-19 affected the profession so far? Has it highlighted/triggered any longer-term changes?)

40. Do you have any other questions or comments you would like to make?
